# Metabolic syndrome and smoking are independent risk factors of male idiopathic infertility

**DOI:** 10.1186/s12610-019-0090-x

**Published:** 2019-07-01

**Authors:** Charlotte Dupont, Céline Faure, Frederic Daoud, Benoit Gautier, Sébastien Czernichow, Rachel Lévy, the ALIFERT collaborative group, Isabelle Aknin, Isabelle Cedrin-Durnerin, Steven Cens, Pascale Chavatte-Palmer, Serge Hercberg, Khaled Pocate, Nathalie Sermondade, Claude Uthurriague, Jean-Philippe Wolf

**Affiliations:** 1 Sorbonne Université, Saint Antoine Research center, INSERM équipe Lipodystrophies génétiques et acquisesService de biologie de la reproduction-CECOS, AP-H, Hôpital Tenon, 4 rue de la Chine, 75020 Paris, France; 2 AP-HP, Hôpital Tenon, service de biologie de la reproduction CECOS, Paris, France; 3 Université Paris Descartes, Paris, France; 4 APHP, Service de nutrition, Hôpital européen Georges-Pompidou, Paris, France

**Keywords:** Metabolic syndrome, Male fertility, Smoking, Syndrome métabolique, Fertilité masculine, Tabagisme

## Abstract

**Background:**

Overweight and obesity are known to impact male fertility and are commonly associated with abdominal obesity and metabolic disorders. The association between abdominal obesity or metabolic syndrome with male reproduction has not been fully investigated. Moreover, many factors may interfere with the evaluation of the impact of metabolic syndrome on male fertility. Thus, tobacco is known to alter the spermatic parameters and phenomena linking smoking with metabolic syndrome are therefore complex. The main objective of this study has been to investigate the potential association of metabolic syndrome with male idiopathic infertility given smoking status.

**Materiel and methods:**

The data of this study concerned infertile (*n* = 96) and fertile (*n* = 100) men under 45 years of age who have been recruited in the ALIFERT case-control study. Body mass index and waist circumference were measured. Serum triglycerides, cholesterol (total, high density lipoprotein, and low density lipoprotein cholesterol) and fasting blood glucose were assayed. Metabolic syndrome has been diagnosed in the presence of at least three of the following criteria: increased waist circumference, high triglycerides, fasting glucose or arterial blood pressure and low high density lipoprotein cholesterol.

**Results:**

The present study reports that infertile men are in poorer health condition compared to fertile men and are more often smokers. The results of this study suggested metabolic syndrome and smoking to be independent risk factor for idiopathic infertility.

**Conclusions:**

Metabolic syndrome and smoking should systematically be checked at the beginning of medical care in infertile males and personal and multifaceted coaching should be proposed to deal jointly with smoking and metabolic disorders.

**Trial registration:**

NCT01093378 ALIFERT. Registered: March 25, 2010.

## Résumé

**Contexte général:**

Le surpoids et l’obésité sont connus pour avoir un impact sur la fertilité masculine et sont souvent associés à l’obésité abdominale et aux désordres métaboliques. L’association entre l’obésité abdominale ou le syndrome métabolique et les fonctions de reproduction masculine n’est. pas totalement élucidé. De plus, de nombreux facteurs confondants peuvent interférer avec la fertilité masculine. Ainsi, le tabac est. connu pour altérer les paramètres spermatiques et des mécanismes complexes lient le tabagisme au syndrome métabolique. L’objectif principal de cette étude est. d’étudier l’association potentielle entre le syndrome métabolique et l’infertilité idiopathique masculine, compte tenu du statut tabagique.

**Matériel et méthodes:**

Les données de cette étude portent sur des hommes de moins de 45 ans infertiles (*n* = 96) et fertiles (*n* = 100) qui ont été recrutés dans le cadre de l’étude cas-témoins ALIFERT. L’indice de masse corporelle et le tour de taille ont été mesurés. Les triglycérides sériques, le cholestérol (total, high density lipoprotein, low density lipoprotein) et la glycémie à jeun ont été analysés. Le syndrome métabolique a été diagnostiqué en présence d’au moins trois des critères suivants: un tour de taille accru, des triglycérides élevés, une glycémie à jeun ou une tension artérielle élevée et un faible taux de cholestérol « high density lipoprotein ».

**Résultats:**

Cette étude rapporte que les hommes infertiles sont en moins bonne santé que les hommes fertiles et sont plus souvent des fumeurs. Les résultats de cette étude suggèrent que le syndrome métabolique et le tabagisme sont des facteurs de risque indépendants de l’infertilité idiopathique.

**Conclusions:**

Le syndrome métabolique et le tabagisme devraient être systématiquement contrôlés au début des soins médicaux chez les hommes infertiles et un accompagnement personnel et multidisciplinaire devrait être proposé pour traiter conjointement le tabagisme et les troubles du métabolisme.

## Introduction

The decline of sperm parameters has been noticed by health care professionals for several years. A meta-analysis has shown that sperm concentration decreased by 52.4% between 1973 and 2011 [1]. It has been hypothesized that this reduction could be associated with the environment and various factors that affect lifestyle, such as exposure to pollution, radiation, high temperatures, and environmental toxicants. Endocrine disrupters in the air, in food, in drinking water, and in cosmetics have also been associated with sperm quality alterations [2]. Furthermore, lifestyle factors that may compromise male gamete production include tobacco, inadequate diet, lack of physical activity, overweight, obesity, and metabolic syndrome [3]. In particular, the association between changes in sperm characteristics and lifestyle factors is of interest because of the reversibility of those factors and the possibility of acting upon them [4].

Overweight and obesity are documented health concerns worldwide, and cohort studies have linked excess body mass index (BMI) with infertility in both males and females [5]. Several studies [6–8] and a meta-analysis [9] have highlighted semen parameter alterations, especially the decrease in sperm count in overweight and obese subjects. An association between obesity and altered sperm DNA integrity has also been suspected [10].

Given its association with fat accumulation, BMI has commonly been used as a proxy to assess body corpulence, but BMI has traditionally not allowed for the discrimination between subcutaneous and visceral fat. The location of body fat has also been shown to be associated with the development of comorbidities in obese individuals. Therefore, excess fat in specific locations of the body might be a more accurate marker of disease than overall BMI [11]. Abdominal obesity is one component of the metabolic syndrome. Metabolic syndrome is defined as the combination of at least three factors including high waist circumference, high triglycerides, high glycaemia, high blood pressure, and low high-density lipoprotein (HDL) cholesterol [12]. The association between abdominal obesity or metabolic syndrome and male reproduction has not been fully investigated to date, but negative associations with reproductive hormones and semen have been previously suggested, although not proven [3, 13–15]. Some studies have reported an impact of the metabolic syndrome on sperm parameters or hormone balance [16]. Nevertheless, there remains controversy [17], which may partly be explained by the heterogeneity of the phenotypes of the metabolic syndrome and its different definitions.

Smoking has been demonstrated to have detrimental effects on human health [18]. Besides cardiovascular and carcinogenic effects, smoking also challenges both male and female reproductive functions [19, 20]. In males, several studies and a meta-analysis have shown a negative association of smoking with semen parameters [21, 22], but less is known about a potential association with male fertility [23].

Although weight gain after smoking cessation has also been reported [24, 25], a few studies have indicated that smoking increases the risk of metabolic syndrome and abdominal obesity through various mechanisms [26–28]. Smoking has been associated with higher body weight and metabolic disorders [29]. In addition, smoking cessation may improve metabolic parameters [26]. Phenomena linking smoking with metabolic syndrome are therefore complex.

The main objective of this study was to investigate the potential association of metabolic syndrome with male idiopathic infertility given smoking status.

## Materials and methods

### Patients

The data of this study concerned infertile (*n* = 96) and fertile (*n* = 100) men younger than 45 years of age who were recruited in the ALIFERT case-control study between September 2009 and December 2013 [30]. The ALIFERT study was designed to assess the association between lifestyle factors and idiopathic infertility in couples (National biomedical research ID no. P071224; ethics committee approval (‘Comité de Protection des Personnes’) ID no. AOM 2009-A00256–51; NEudra CT ID no. 08180; clinicaltrials.gov ID no. NCT01093378).

Idiopathic or unexplained infertility may be defined by a lack of diagnosis made in couples who failed to conceived after one or two years of unprotected sexual intercourse [31]. It may impact 30 to 40% of infertile couples [32]. Standard investigation protocol does not provide a diagnosis to explain the infertility. In men, semen analysis is usually performed. It is important to study idiopathic infertility, indeed: even if no cause is clearly identified, the environment and lifestyle could explain it. A better understanding of the origin of the disorder should help in managing infertile couples.

The study group consisted of infertile male partners of couples attending four infertility centres in France, as follows: the Jean Verdier Hospital assisted reproductive Technology (ART) centre, Bondy; the Cochin Hospital ART centre, Paris; the Hôpital Nord ART centre, Saint Etienne; and the Polyclinique de Navarre ART centre, Pau. Study participants were recruited after an initial infertility check-up. Semen analysis for conventional parameters and a semen culture were performed. Medical examination was performed if necessary and medical history was reported. Subject eligibility criteria were (1) primary idiopathic infertility lasting longer than 12 months; (2) age younger than 45 years; (3) no severe oligozoospermia (< 5 million/mL); (4) no alterations of the male reproductive organs such as undescended testis, varicocele, or infection; (5) no female factors of infertility; (6) a female partner age of younger than 38 years; and (7) the provision of written informed consent.

The control group consisted of fertile male volunteers recruited from the general healthy population in the areas of the participating centres. Inclusion criteria for this group were (1) a spontaneously conceived child under two years of age, (2) time to pregnancy of less than 12 months, (3) age younger than 45 years, (4) a female partner age of younger than 38 years, and (5) the provision of written informed consent. Members of the control group did not undergo clinical examination or semen parameter analysis before inclusion; however, they were asked about their medical history.

All participants were Caucasians. Infertile and fertile males with current known or previous metabolic or digestive disease were not included.

### Assessments

All study and control subjects were assessed by the same trained investigator using the same calibrated devices.

### Anthropometric assessments

Height, weight (Tanita BC-420MA analyser), and waist circumference at the narrowest point between the lower border of the ribs and the iliac crest were evaluated.

### Blood samples and analyses

Blood samples were collected after a 12-h fasting period. HDL cholesterol, low-density lipoprotein (LDL) cholesterol, triglycerides, and glucose concentrations were instantly measured by standardized methods after centrifugation in the hospitals’ biology laboratories.

### Blood pressure assessment

Systolic and diastolic blood pressures were measured using a sphygmomanometer cuff around the patient’s arm after five minutes of bed rest in a supine position. The systolic and diastolic blood pressure measurements were the respective means of their right and left values.

### Metabolic syndrome definition

Metabolic syndrome was diagnosed in the presence of at least three of the following criteria: waist circumference of more than 92 cm, triglycerides of 150 mg/dL / 1.7 mmol/L or more, HDL cholesterol of less than 40 mg/dL / 1.0 mmol/L, fasting glucose of 100 mg/dL / 5.6 mmol/L or more, and arterial blood pressure of 130/85 mmHg or higher [12].

### Smoking status

Patients reported the number of cigarettes smoked per day and were categorized as a smoker if they smoked one or more cigarettes per day and as a nonsmoker if they did not smoke at all. Exhaled carbon monoxide (CO) was measured in parts per million (ppm) as a supportive indicator with the underlying assumption that exhaled carbon monoxide (CO) in smokers [33] is higher than that in nonsmokers. Exhaled CO measurement was performed by having subjects exhale completely and then inhale fully in open air, holding their breath for 10 s, and then exhaling completely into a portable CO monitor (Tabataba analyser; FIM Medical, Villeurbanne, France) [33].

### Statistical analyses

Data were summarized using means and standard deviations. The following test plan was applied: (1) differences between infertile and fertile males (t-test), (2) association between smoking and metabolic syndrome (Fisher’s exact test), and (3) association between fertility as a dependent variable and both metabolic syndrome and smoking as independent variables (logistic regression). Bonferroni correction was applied to cap overall type I error to 5% when comparing fertility, metabolic syndrome, and smoking, respectively, between the two groups, thus resulting in a type I error of 1.7% per test.

## Results

### Population description

No statistical differences were found between infertile and fertile males in term of age, total cholesterol, LDL cholesterol, and triglyceride levels. Also, blood pressures were not significantly different.

However, significant differences did include a higher BMI in the infertile group versus the control group [25.9 kg/m^2^, 95% confidence interval (CI): 25.0–26.7 vs. 23.9, 95% CI: 23.4–24.5; *p* = 0.00020] as well as a higher waist circumference (91.5 cm, 95% CI: 89.3–93.8 vs. 86.1, 95% CI: 84.7–87.6; *p* < 0.0001), a higher fasting blood glucose (5.0 mmol/L, 95% CI: 4.8–5.1 vs. 4.3, 95% CI: 4.1–4.5; p < 0.0001), and a lower HDL cholesterol levels (1.25 mmol/L, 95% CI: 1.18–1.33 vs. 1.38 mmol/L, 95% CI: 1.32–1.44; *p* < 0.008) (Table 1).

**Table 1:** Comparison of anthropometric, metabolic parameters and exhaled CO between subfertile and fertile men

	Subertile (*n* = 96)	Fertile (*n* = 100)	*p*
Age	33.3[32.2, 34.3]	34.4 [33.7, 35.1]	NS
BMI (Kg.m-2)	25.9 [25.0, 26.7]	23.9 [23.4, 24.5]	0.0002
Waist circumference (cm)	91.5 [89.3, 93.8]	86.1 [84.7, 87.6]	0.0001
Fasting blood glucose (mmol/l)	5.0 [4.8, 5.1]	4.3 [4.1, 4.5]	< 0.0001
Total cholesterol (mmol/l)	5.2 [5.01, 5.40]	5.2 [5.02, 5.38]	NS
HDL cholesterol (mmol/l)	1.25 [1.18, 1.33]	1.38 [1.32, 1.44]	< 0.008
LDL cholesterol (mmol/l)	3.28 [3.09, 3.47]	3.24 [3.06, 3.41]	NS
Triglycerides (mmol/l)	1.42 [1.23, 1.62]	1.20 [1.06, 1.33]	NS
Systolic blood pressure (mmHg)	126 [124, 129]	126 [124, 128]	NS
Diastolic blood pressure (mmHg)	81 [79, 83]	80 [79, 82]	NS

Data reported as mean and 95% CI (confidence interval). BMI (body mass index), HDL (high density lipoprotein), LDL (low density lipoprotein)

### Metabolic syndrome and smoking

Both metabolic syndrome and smoking were found to be more frequent in infertile than in fertile males (Table 2), as follows: metabolic syndrome [17/95 (17.9, 95% CI: 10.8–27.1%) vs. 6/99 (6.1, 95% CI: 2.3–12.7%); Fisher’s exact test *p* = 0.014] and smoking [27/94 (28.7, 95% CI: 19.9–39.0%) vs. 14/99 (14.1, 95% CI: 8.0–22.6%); Fisher’s exact test p = 0.014]. Smoking status was not significantly different between males with vs. without metabolic syndrome [7/22 (31.8%, 95 CI: 13.9–54.9%) vs. 34/170 (20, 95% CI: 14.3–26.8%); Fisher’s exact test *p* = 0.27].

**Table 2:** Proportion of men with metabolic syndrome and smokers between the groups of subfertile and fertile men

	Subertile (n = 96)	Fertile (*n* = 99)	p
Metabolic syndrome (%, CI)	17.9 [10.0, 25.7]	6.1 [1.3, 10.8]	0.0115
Smokers (%, CI)	28.7 [19.4,38.0]	14.1 [7.2, 21.1]	0.014

CI (confidence interval)

Logistic regression confirmed the absence of an interaction between metabolic syndrome and smoking.

Exhaled CO was significantly higher in smokers (9.2 ppm, 95% CI: 7.2–11.1) than in nonsmokers (3.4 ppm, 95% CI: 3.1–3.7; *p* < 0.0001).

## Discussion

The strengths of the present study are: (1) comparable infertile and fertile control groups, with respect to most variables other than the study variables; (2) the availability of data in the fertile control group, and (3) assessments by the same trained investigator using the same calibrated devices so as to minimize observation bias. A weakness is the fact that the study sample was identified through a demand for medical assistance, whereas the control population was not, thus causing potential participation bias.

Another limitation was the self-reporting of tobacco consumption, which may be underestimated by some men. The assessment of exhaled CO was intended to support self-reporting, but exhaled CO can be influenced by passive smoking and prolonged exposure to a polluted environment [34] so that the level of exhaled CO does not necessarily accurately reflect the amount of smoking done by a subject.

Previous studies have separately analysed the association between semen parameters and abdominal obesity or metabolic syndrome on the one hand and between semen parameters and smoking on the other. Many of these studies suggested a negative impact of abdominal obesity or metabolic syndrome on semen parameters [3, 13, 14]. However, they did not assess male fertility. Other studies have highlighted the negative impact of smoking on semen parameters [21].

The mechanisms involved in these phenomena are complex and multifactorial. Overweightness and obesity have been known for a long time to have a negative impact on conventional and nonconventional semen parameters [35]. Hormonal disorders, systemic inflammation, oxidative stress, and scrotal temperature increase related to obesity are involved in the observed effects. However, it is important to consider the comorbidities associated with obesity, which can also play a critical role. For example, diabetes mellitus may alter semen parameters and male fertility through various mechanisms [36]. There is more evidence that obesity, metabolic disorders, and diabetes mellitus decrease serum testosterone levels. This action may be associated with a defect in Leydig cells [36]. Furthermore, obesity and metabolic disorders are associated with a decrease in androgen production and an increase in aromatase activity as linked to excess adipose tissue. Insulin resistance is responsible for a decrease in sex hormone–binding globulin levels. The consequences are a decrease in plasma testosterone levels and an increase in estradiolemia. Feedback from the hypothalamic–pituitary–gonadal axis is therefore disrupted, leading to hypogonadism. The functions of Sertoli cells are therefore impacted and sperm production is altered.

The originality of the present case-control study was to jointly compare anthropometric and metabolic parameters and smoking in the male partners of idiopathic infertile couples and fertile couples, respectively. The results of this study suggest metabolic syndrome and smoking to be independent risk factors for idiopathic infertility.

Few studies so far have assessed the effect of smoking cessation on semen parameters, hormonal status [37], male fertility, and changes in sexual dysfunction [38, 39].

Nicotine may have a direct negative effect on sperm quality [40], and smoking is understood to alter sperm DNA integrity and induce sperm apoptosis [41].

The results of these previous studies support the need to encourage smoking cessation among infertile men. However, it is important to anticipate weight gain after tobacco cessation [42], given that weight gain can be associated with metabolic disorders and overweightness and obesity, which are known to impact male fertility. While previously published studies found an increased risk of metabolic syndrome in active smokers, the current study was not able to find a significant effect, although we found nonsignificantly identified higher rates of metabolic syndrome in smokers [26, 27].

It is widely recognized that nutritional management and increased physical activity can improve cardiovascular comorbidities associated with the metabolic syndrome [43], but to our knowledge no data are available about the impact of lifestyle interventions on reproductive functions in the case of metabolic disorders or metabolic syndrome.

Several studies have shown an improvement in the hormonal balance and erectile function of obese patients after weight loss [44–46]. However, the impact of weight loss on sperm parameters has only been investigated in a limited fashion to date. An improvement in sperm parameters after weight loss has been reported in two studies [45, 47]. Additionally, it has been previously noted in six men with abdominal obesity that abdominal fat loss led to an improvement of sperm DNA integrity linked to an oxidative stress decrease and an antioxidant protection increase in seminal plasma. All female partners became pregnant following the lifestyle intervention [4].

The results of our study suggest that personal and multifaceted coaching should be proposed to deal jointly with smoking and metabolic disorders through nutritional improvement, physical activity, and treatments for high blood pressure and diabetes mellitus. The completion of further prospective interventional randomized controlled trials would be relevant to quantify the benefit of such an approach.

For greater efficiency, young men should be informed about the effects of metabolic syndrome and smoking on fertility as soon as possible. They should strive to improve their lifestyle before they face difficulties with conceiving a child.

Others environmental effects that were not assessed in this study are of importance and may challenge male fertility. Poor lifestyle can also be associated with excessive alcohol consumption, and this can have repercussions on male fertility [48]. Furthermore, men with metabolic disorders and, in particular, dyslipidaemia, may be treated with statins that may reduce testosterone levels and interfere with reproductive functions [49]. Finally, exposure to other environmental toxins such as pollution or mobile phone radiation [50] may be confounding factors.

## Conclusion

The present study reports that infertile men demonstrate a poorer health condition as compared with fertile men and are more often smokers. Metabolic syndrome and tobacco in men appear as significant and independent risks factors for idiopathic infertility. Health care professionals should be aware of this phenomenon and risks factors should systematically be checked at the beginning of medical care, considering that adverse consequences may be reversible or even preventable.

## Abbreviations

ART: Assisted reproductive technology
BMI: Body mass index
CI: Confidence interval
HDL: High density lipoprotein
LDL: Low density lipoprotein
